# Association of monocyte to high-density lipoprotein cholesterol ratio with non-calcified plaques in subclinical coronary atherosclerosis: a cross-sectional study in hypertensive patients

**DOI:** 10.3389/fendo.2026.1941868

**Published:** 2026-09-17

**Authors:** Xuekang Su, Shuying Wu, Shuangshuang Xue, Peiying Chen, Weichao Wei, Yanghui Gu, Qiang Liu, Mingtai Chen, Yingnan Chen, Tao Li, Xiaojuan Lin, Chong Xu, Zhicong Zeng, Biao Li

**Affiliations:** 1 The Fourth Clinical Medical College of Guangzhou University of Chinese Medicine, Shenzhen, Guangdong, China; 2 Department of Cardiology, Shenzhen Traditional Chinese Medicine Hospital, Shenzhen, Guangdong, China; 3 The Seventh Clinical College of Guangzhou University of Chinese Medicine, Shenzhen, Guangdong, China; 4 Department of Cardiology, Shenzhen Bao'an Chinese Medicine Hospital, Guangzhou University of Chinese Medicine, Shenzhen, Guangdong, China

**Keywords:** biomarker, hypertension, inflammation, MHR, non-calcified plaque, subclinical coronary atherosclerosis

## Abstract

**Background:**

Hypertension is a major driver of atherosclerotic cardiovascular disease (ASCVD), many hypertensive patients harbor occult subclinical coronary atherosclerosis (SCA). The monocyte to high-density lipoprotein cholesterol ratio (MHR), a novel biomarker integrating inflammation and lipid metabolism, has emerged as a potential risk indicator. However, its value in identifying coronary artery characteristics in asymptomatic hypertensive populations remains unclear. We aimed to investigate the association between MHR and subclinical coronary atherosclerosis characteristics in asymptomatic hypertensive patients.

**Methods:**

We conducted a cross-sectional study enrolling 283 hypertensive patients who underwent coronary computed tomography angiography (CCTA) at Shenzhen Traditional Chinese Medicine Hospital between January 2019 and September 2024. Patients were categorized into quartiles (Q1-Q4) based on MHR levels. CCTA was used to evaluate coronary artery calcium score (CACS) and plaque characteristics (non-calcified, calcified, mixed). Multivariable logistic regression, restricted cubic spline (RCS), and receiver operating characteristic (ROC) curve analyses were performed.

**Results:**

There was a significant difference in the prevalence of non-calcified plaques across MHR quartiles (*P* < 0.001). After fully adjusting for confounders including age, gender, smoking, BMI, TG, LDL-C, serum creatinine, statin use and diabetes, patients in the highest MHR quartile (Q4) had a 5.97-fold higher risk of having non-calcified plaques compared to those in Q1 (OR = 5.97, 95% CI: 2.63-14.1, *P* < 0.001; *P* for trend < 0.001). RCS analysis revealed a significant non-linear positive association between MHR and non-calcified plaque risk (*P* for nonlinearity = 0.007), characterized by an initial increase, a subsequent transient decline, and a marked rise at higher MHR values. The area under the ROC curve (AUC) for MHR in identifying non-calcified plaques was 0.700. Subgroup analyses revealed no significant interaction between MHR and age, sex, diabetes, smoking status, BMI category, or statin use (*P* for interaction > 0.05).

**Conclusion:**

Elevated MHR is independently and non-linearly associated with the presence of non-calcified plaques in hypertensive patients. Given that non-calcified plaques represent an active, lipid-driven inflammatory state, MHR may serve as a simple and cost-effective adjunctive biomarker to help identify individuals with a higher burden of such plaques in asymptomatic hypertensive patients. However, its role in clinical risk stratification requires validation in prospective studies.

## 1 Introduction

Hypertension is a major risk factor for atherosclerotic cardiovascular disease (ASCVD). Prolonged hypertension can lead to damage and remodeling of the vascular walls. A substantial proportion of hypertensive patients remain entirely asymptomatic, lacking typical angina pectoris, yet may already harbor subclinical coronary atherosclerosis (SCA). Even without overt symptoms, subclinical coronary atherosclerosis confers significant cardiovascular risk. Subclinical obstructive disease is associated with an over 8-fold increased risk of myocardial infarction (1) and atherosclerotic burden independently predicting all-cause mortality (2). Consequently, this “silent window” carries substantial prognostic weight, early identification of high-risk individuals during this preclinical phase is imperative for effective cardiovascular prevention.

The pathogenesis of atherosclerosis is fundamentally driven by the interplay between lipid metabolic disorders and chronic low-grade inflammation (3). Circulating monocytes initiate and propagate vascular inflammation by adhering to activated endothelium, migrating into the intima, differentiating into macrophages, and engulfing oxidized low-density lipoprotein to form lipid-laden foam cells—the hallmark of early atheroma (4–6). Conversely, high-density lipoprotein cholesterol (HDL-C) exerts vasculoprotective effects through reverse cholesterol transport, anti-inflammatory, and antioxidant mechanisms (7, 8). Therefore, the monocyte-to-HDL-C ratio (MHR) represents a pathophysiological ‘tug-of-war’ between pro-inflammatory/pro-oxidant pathways and anti-inflammatory/antioxidant defense mechanisms. Compared with monocyte count or HDL-C alone, MHR more sensitively captures vascular inflammatory-metabolic imbalance, offering superior reflection of the pathological interplay between inflammatory burden and compromised HDL-mediated protection.

Emerging evidence consistently associates elevated MHR with adverse cardiovascular outcomes and coronary artery disease severity in symptomatic populations (9–12), but its relationship to specific lesion characteristics in asymptomatic hypertensive individuals remains unclear. This study aims to investigate the independent association between MHR and SCA phenotypes, with particular emphasis on plaque composition and calcification burden as assessed by coronary computed tomography angiography (CCTA), thereby establishing a readily available biomarker for the identification of early atherosclerotic phenotypes in asymptomatic hypertensive patients.

## 2 Methods

### 2.1 Study design and population

This retrospective cross-sectional study was conducted at Shenzhen Traditional Chinese Medicine Hospital.

Given the retrospective cross-sectional design, conventional prospective sample size calculation was not performed. Instead, we assessed the statistical adequacy of the multivariable logistic regression models using the Events Per Variable (EPV) guideline. The fully adjusted model included 10 independent variables (MHR quartiles as the exposure and 9 covariates: age, sex, smoking, BMI, triglycerides, LDL-C, serum creatinine, statin use, and diabetes). Based on the EPV criterion of ≥10 events per variable, a minimum of 100 outcome events was required for stable parameter estimation. The prespecified primary outcome, non-calcified plaque, occurred in 124 patients, satisfying this threshold. Other evaluated outcomes, including any plaque (n = 182) and moderate-to-severe calcification (n = 219), also met the EPV criterion. Thus, the final sample of 283 patients was considered adequate for the planned multivariable analyses.

We consecutively reviewed 283 hypertensive patients who underwent coronary computed tomography angiography (CCTA) between January 2019 and September 2024.

Inclusion criteria were: (1) Age 18–80 years; (2) Diagnosis of hypertension (defined as systolic blood pressure ≥140 mmHg and/or diastolic blood pressure ≥90 mmHg on three separate occasions without antihypertensive medication, or documented history of hypertension with current antihypertensive therapy); (3) Underwent CCTA with diagnostic image quality adequate for plaque assessment; (4) Complete data available for the exposure variables (monocyte count and HDL-C, allowing calculation of MHR) and outcome variables (CCTA-assessed coronary plaque characteristics, including CACS and plaque composition).

Exclusion criteria were: (1) Prior diagnosis of coronary artery disease, including stable or unstable angina pectoris, prior myocardial infarction, percutaneous coronary intervention (PCI), or coronary artery bypass grafting (CABG); (2) Acute or chronic infection; (3) Severe hepatic dysfunction (documented cirrhosis, hepatic failure, alanine aminotransferase or aspartate aminotransferase >3 times the upper limit of normal, or total bilirubin >2 times the upper limit of normal) or severe renal dysfunction (estimated glomerular filtration rate <30 mL/min/1.73 m² or need for renal replacement therapy); (4) Malignancy; (5) Autoimmune disease; (6) Cerebrovascular events (stroke or transient ischemic attack) or aortic dissection within the preceding 6 months; (7) Pregnancy or lactation.

### 2.2 Data collection and biochemical measurements

Demographic characteristics and clinical data were extracted from electronic medical records, including age, sex, body mass index (BMI), smoking status, systolic and diastolic blood pressure, statin use, and comorbidities (diabetes mellitus). Other common cardiovascular risk factors, such as obesity, dyslipidemia, and renal function, were quantitatively evaluated using continuous clinical and laboratory measurements.

Fasting venous blood samples were collected for biochemical analyses, including complete blood count, lipid profile [total cholesterol (TC), triglycerides (TG), low-density lipoprotein cholesterol (LDL-C), and HDL-C], fasting blood glucose (FBG), glycated hemoglobin (HbA1c), serum creatinine, and uric acid. Biochemical parameters were measured using standard automated methods on clinical chemistry analyzers: enzymatic colorimetric assay for triglycerides, cholesterol oxidase method for total cholesterol, selective inhibition method for HDL-C, soluble reaction method for LDL-C, hexokinase method for fasting glucose, enzymatic method for creatinine, uricase ultraviolet method for uric acid, and boronate affinity high-performance liquid chromatography for HbA1c. The complete blood count, including monocyte enumeration, was determined using automated hematology analyzers based on fluorescent flow cytometry (WDF channel).

The monocyte-to-HDL-C ratio (MHR) was calculated using the formula: MHR = monocyte count (×10^9^/L)/HDL-C (mmol/L).

### 2.3 CCTA acquisition and analysis

CCTA was performed using a Siemens SOMATOM Force dual-source CT scanner following standard protocols. CCTA images were analyzed independently by experienced radiologists blinded to clinical data according to the Coronary Artery Disease Reporting and Data System (CAD-RADS) 2.0 criteria. Coronary artery calcium score (CACS) was calculated using the Agatston method.

Plaque characterization: Plaques were classified into three categories based on CT attenuation values: calcified plaque: CT value >130 HU, appearing as high-density with well-defined borders; non-calcified plaque: CT value <60 HU, appearing as low-density with ill-defined borders, indicating lipid-rich or fibrous components; mixed plaque: Containing both calcified and non-calcified components.

Outcome definitions: significant stenosis: Luminal diameter reduction ≥50% in any coronary artery segment; moderate-to-severe calcification: CACS >100; any plaque: presence of any coronary plaque (calcified, non-calcified, or mixed) regardless of stenosis severity. Notably, calcified plaque refers to a lesion-level morphological phenotype identified on CCTA (CT value >130 HU), reflecting the composition of an individual atherosclerotic lesion. In contrast, moderate-to-severe calcification (CACS >100) is a patient-level aggregate score quantifying the global coronary calcium burden across the entire coronary tree. These two metrics capture distinct pathological constructs and were analyzed separately.

### 2.4 Statistical analysis

Analyses were performed using R software (v4.4.2). Only BMI (82 missing, 28.98%) and HbA1c (62 missing, 21.91%) contained missing data; all other clinical, laboratory, and outcome variables were fully observed. Missing data in BMI and HbA1c were handled using Multiple Imputation by Chained Equations (MICE), with Classification and Regression Trees (CART) and Predictive Mean Matching (PMM) as the imputation methods; these approaches are robust against multicollinearity. MICE was not applied to other variables as they had no missing values. Continuous variables were compared using ANOVA or Kruskal-Wallis tests; categorical variables using Chi-square tests. The continuous MHR variable was categorized into quartiles (Q1–Q4) to facilitate intuitive comparison of coronary lesion risk across different MHR levels and to examine the potential dose-response relationship with the outcome. Multivariable logistic regression was used to evaluate the association between MHR and SCA. Covariates for the multivariable models were selected based on clinical relevance, prior literature, and statistical significance in univariable analysis (*P* < 0.10). Highly correlated redundant variables were strictly excluded to avoid multicollinearity. The fully adjusted model (Model 3) included age, gender, smoking, BMI, TG, LDL-C, SCR, statin use, and diabetes. Restricted cubic spline (RCS) analysis was performed using the rms package in R, with 4 knots placed at the default quantile positions (5th, 35th, 65th, and 95th percentiles) of the MHR distribution. The RCS model was adjusted for the same covariates as the fully adjusted logistic regression model (Model 3). ROC curves assessed diagnostic performance. Subgroup analyses were performed using standardized MHR (Z-score), with interaction tests. *P* < 0.05 was considered statistically significant.

## 3 Results

### 3.1 Baseline characteristics of the study population

A total of 283 participants (153 men, 54.0%) with a mean age of 57.26 ± 11.66 years were enrolled. All participants were divided into four groups based on MHR quartiles. The median MHR values were 0.26 (0.24, 0.29), 0.38 (0.34, 0.40), 0.47 (0.45, 0.50), and 0.63 (0.57, 0.71) in groups Q1 (lowest), Q2, Q3, and Q4 (highest), respectively. Age differed significantly among the four groups (*P* = 0.005), with Q4 having the lowest mean age and Q2 having the highest. Sex distribution and smoking status also differed significantly among the groups (both *P* < 0.001); the proportion of females decreased from 66% in Q1 to 21% in Q4, whereas the proportion of males increased from 34% to 79%, and smoking prevalence rose from 11% in Q1 and Q2 to 47% in Q4. HDL-C, triglycerides, and serum creatinine levels differed significantly among the quartiles (all *P* < 0.01). HDL-C was highest in Q1 and lowest in Q4, whereas triglycerides were lowest in Q1 and highest in Q4. Serum creatinine showed a similar pattern, with the highest median value observed in Q3. There was no significant difference in the prevalence of diabetes history among the four groups. BMI, systolic and diastolic blood pressure, total cholesterol, LDL-C, fasting glucose, HbA1c, statin use, and uric acid did not differ significantly among the groups. **Table 1** shows the clinical characteristics of the study cohort.

**Table 1:** Clinical characteristics of the study cohort.

Characteristic	Overall (N=283)	Q1 N = 71	Q2 N = 71	Q3 N = 71	Q4 N = 70	P
Age (years)	57.26 ± 11.66	58.59 ± 10.35	59.45 ± 11.50	57.82 ± 12.64	53.11 ± 11.24	0.005
Sex						<0.001
Female n (%)	130 (46%)	47 (66%)	45 (63%)	23 (32%)	15 (21%)	
Male n (%)	153 (54%)	24 (34%)	26 (37%)	48 (68%)	55 (79%)	
BMI (kg/m²)	25.39 (23.53, 27.68)	25.39 (23.38, 27.12)	25.25 (23.11, 28.25)	25.47 (23.78, 27.76)	25.56 (23.38, 27.68)	0.928
Systolic BP (mmHg)	141.94 ± 18.97	142.07 ± 20.11	140.89 ± 16.80	140.28 ± 18.91	144.56 ± 20.01	0.575
Diastolic BP (mmHg)	88.00 (79.00, 98.00)	88.00 (80.00, 97.00)	85.00 (79.00, 94.00)	89.00 (77.00, 98.00)	90.00 (81.00, 102.00)	0.411
Smoking Status n (%)	74 (26%)	8 (11%)	8 (11%)	25 (35%)	33 (47%)	<0.001
Diabetes History n (%)	87 (31%)	17 (24%)	22 (31%)	22 (31%)	26 (37%)	0.409
Triglycerides (mmol/L)	1.50 (1.05, 2.23)	1.24 (0.92, 1.78)	1.48 (1.05, 2.23)	1.50 (1.09, 2.37)	1.79 (1.26, 2.60)	0.002
Total Cholesterol (mmol/L)	4.63 (3.89, 5.33)	4.71 (4.12, 5.27)	4.68 (3.89, 5.53)	4.48 (3.64, 5.48)	4.51 (3.92, 5.27)	0.591
HDL-C (mmol/L)	1.11 (0.95, 1.30)	1.32 (1.14, 1.44)	1.15 (0.99, 1.33)	1.08 (0.95, 1.26)	0.96 (0.81, 1.07)	<0.001
LDL-C (mmol/L)	2.97 (2.24, 3.61)	3.16 (2.37, 3.62)	2.93 (2.28, 3.71)	2.84 (2.07, 3.59)	2.94 (2.25, 3.46)	0.795
Fasting Glucose (mmol/L)	5.14 (4.66, 6.00)	5.09 (4.71, 5.68)	5.06 (4.61, 5.85)	5.22 (4.74, 5.88)	5.18 (4.48, 6.45)	0.928
HbA1c (%)	6.20 (5.70, 7.40)	6.20 (5.80, 7.40)	6.00 (5.70, 7.20)	6.10 (5.60, 6.90)	6.35 (5.70, 7.70)	0.745
Serum Creatinine (μmol/L)	74.00 (61.00, 88.00)	67.00 (59.00, 79.00)	73.00 (58.00, 89.00)	82.00 (63.00, 90.00)	79.00 (64.00, 95.00)	0.002
Uric Acid (μmol/L)	350.00 (297.00, 418.00)	340.00 (304.00, 395.00)	337.00 (279.00, 409.00)	358.00 (303.00, 420.00)	378.50 (301.00, 447.00)	0.094
MHR	0.42 (0.32, 0.53)	0.26 (0.24, 0.29)	0.38 (0.34, 0.40)	0.47 (0.45, 0.50)	0.63 (0.57, 0.71)	<0.001

Values are given as the mean ± standard deviation, median (interquartile range), or number n (%), P-value of < 0.05 was considered significant. BMI, Body mass index; BP, Blood pressure; HbA1c, Hemoglobin A1c, HDL-C, High-density lipoprotein cholesterol, LDL-C, Low-density lipoprotein cholesterol; MHR, Monocyte to high-density lipoprotein cholesterol ratio.

### 3.2 Prevalence of coronary artery characteristics across MHR quartiles

The prevalence of any plaque varied significantly across MHR quartiles, rising from 51% in Q1 to 73% in Q4 (*P* = 0.038). The prevalence of non-calcified plaque also differed significantly among quartiles, increasing from 21% in Q1 to 69% in Q4 (*P* < 0.001). Similarly, the proportion of patients with moderate-to-severe calcification (CACS > 100) varied across quartiles, from 63% in Q1 to 87% in Q4 (*P* = 0.007). In contrast, the prevalence of calcified plaques, mixed plaques, and significant stenosis did not differ significantly across the groups, suggesting a specific propensity for MHR to associate with lipid-rich, non-calcified atherosclerotic burdens. The prevalence of coronary artery characteristics across MHR quartiles is presented in **Table 2**.

**Table 2:** The prevalence of coronary artery characteristics across MHR quartiles.

Characteristic	Overall (N=283)	Q1 N = 71	Q2 N = 71	Q3 N = 71	Q4 N = 70	P
Moderate-Severe Calcification	219 (77%)	45 (63%)	57 (80%)	56 (79%)	61 (87%)	0.007
Any Plaque	182 (64%)	36 (51%)	47 (66%)	48 (68%)	51 (73%)	0.038
Non-calcified Plaque	124 (44%)	15 (21%)	28 (39%)	33 (46%)	48 (69%)	<0.001
Calcified Plaque	99 (35%)	28 (39%)	31 (44%)	21 (30%)	19 (27%)	0.123
Mixed Plaque	61 (22%)	13 (18%)	18 (25%)	14 (20%)	16 (23%)	0.739
Significant Stenosis	39 (14%)	8 (11%)	10 (14%)	12 (17%)	9 (13%)	0.798

### 3.3 Association between MHR and coronary artery characteristics

In the crude model, participants in the highest MHR quartile (Q4) had an 8.15-fold increased risk of non-calcified plaque compared to those in Q1 (OR 8.15, 95% CI 3.89–17.9, *P* < 0.001). Although the associations were somewhat attenuated after adjusting for potential confounders, the trend remained robust and statistically significant. In the fully adjusted Model 3 (adjusted for age, sex, smoking, diabetes, BMI, TG, LDL-C, statin use, SCR), the ORs for Q2, Q3, and Q4 were 2.34 (95% CI 1.09–5.18, *P* = 0.031), 2.45 (95% CI 1.13–5.46, *P* = 0.026), and 5.97 (95% CI 2.63–14.1, *P* < 0.001), respectively, with a consistent increasing trend across quartiles (*P* for trend < 0.001). In the crude model, participants in the highest MHR quartile (Q4) had a 2.61-fold increased risk of any plaque compared to those in Q1 (OR 2.61, 95% CI 1.30–5.34, *P* = 0.007). After full adjustment for potential confounders, this association was attenuated and became marginally significant (Q4 vs. Q1: OR 2.31, 95% CI 1.01–5.41, *P* = 0.049; *P* for trend = 0.057). For moderate-to-severe calcification, the fully adjusted model showed a significant increase in risk for Q4 versus Q1 (OR 2.60, 95% CI 1.03–6.96, *P* = 0.048), but the trend across quartiles did not reach statistical significance (*P* for trend = 0.059). In contrast, no significant associations were observed for calcified plaque, mixed plaque, or significant stenosis after full adjustment (**Table 3**).

**Table 3:** Association between MHR and on coronary artery characteristics.

Characteristic	Model 1 (Unadjusted)			Model 2 (partially adjusted)			Model 3 (fully adjusted)			
	OR	95% CI	P	OR	95% CI	P	OR	95% CI	P	P for Trend
Any Plaque										0.053
Q1	—	—		—	—		—	—		
Q2	1.90	0.97, 3.78	0.062	1.87	0.94, 3.75	0.076	1.71	0.84, 3.53	0.14	
Q3	2.03	1.03, 4.05	0.042	1.73	0.85, 3.56	0.130	1.71	0.81, 3.64	0.20	
Q4	2.61	1.30, 5.34	0.007	2.45	1.16, 5.29	0.020	2.30	1.02, 5.29	0.047	
Non-calcified Plaque										<0.001
Q1	—	—		—	—		—	—		
Q2	2.43	1.17, 5.21	0.019	2.40	1.14, 5.19	0.023	2.39	1.11, 5.27	0.028	
Q3	3.24	1.58, 6.92	0.002	2.60	1.23, 5.65	0.014	2.50	1.16, 5.56	0.022	
Q4	8.15	3.89, 17.9	<0.001	6.61	3.04, 15.0	<0.001	5.91	2.62, 13.9	<0.001	
Calcified Plaque										0.022
Q1	—	—		—	—		—	—		
Q2	1.19	0.61, 2.33	0.60	1.13	0.56, 2.25	0.70	1.07	0.53, 2.19	0.8	
Q3	0.65	0.32, 1.29	0.20	0.48	0.22, 1.02	0.058	0.49	0.22, 1.05	0.070	
Q4	0.57	0.28, 1.16	0.12	0.49	0.22, 1.05	0.070	0.45	0.20, 1.03	0.062	
Mixed Plaque										0.355
Q1	—	—		—	—		—	—		
Q2	1.52	0.68, 3.45	0.30	1.43	0.63, 3.31	0.40	1.21	0.52, 2.89	0.70	
Q3	1.10	0.47, 2.56	0.80	0.84	0.34, 2.04	0.70	0.71	0.28, 1.78	0.50	
Q4	1.32	0.58, 3.04	0.50	1.14	0.47, 2.79	0.80	0.74	0.28, 1.91	0.50	
Moderate-Severe Calcification										0.064
Q1	—	—		—	—		—	—		
Q2	2.35	1.12, 5.13	0.027	2.36	1.11, 5.18	0.028	2.29	1.06, 5.12	0.038	
Q3	2.16	1.03, 4.63	0.044	1.74	0.80, 3.85	0.20	1.66	0.74, 3.77	0.20	
Q4	3.92	1.73, 9.60	0.002	2.88	1.21, 7.34	0.020	2.56	1.02, 6.83	0.051	
Significant Stenosis										0.520
Q1	—	—		—	—		—	—		
Q2	1.29	0.48, 3.59	0.60	1.24	0.45, 3.49	0.70	1.14	0.40, 3.33	0.80	
Q3	1.60	0.62, 4.35	0.30	1.20	0.44, 3.36	0.70	1.19	0.43, 3.42	0.70	
Q4	1.16	0.42, 3.28	0.80	0.85	0.29, 2.54	0.80	0.67	0.21, 2.11	0.50	

P-value of < 0.05 was considered significant. MHR, Monocyte-to-HDL ratio; CI, Confidence interval; OR, Odds ratio; BMI, Body mass index; TG; Triglycerides, LDL-C, Low-density lipoprotein cholesterol; SCR, Serum creatinine. Model 1: Unadjusted; Model 2: Adjusted for age, sex; Model 3: Fully adjusted for age, sex, smoking, diabetes, BMI, TG, LDL-C, statin use, and SCR.

### 3.4 Diagnostic value and dose-response relationship

ROC curve analysis was performed to evaluate the diagnostic utility of MHR (**Figure 1**, **Table 4**). The area under the curve (AUC) for predicting non-calcified plaque was 0.700 (95% CI 0.640–0.761, *P* < 0.001), with an optimal cutoff value of 0.423, yielding a sensitivity of 65.3% and a specificity of 64.2%. For any plaque, the AUC was 0.603 (95% CI 0.534–0.672, *P* = 0.004), with an optimal cutoff value of 0.343, yielding a sensitivity of 74.2% and a specificity of 43.6%.

**Figure 1:**
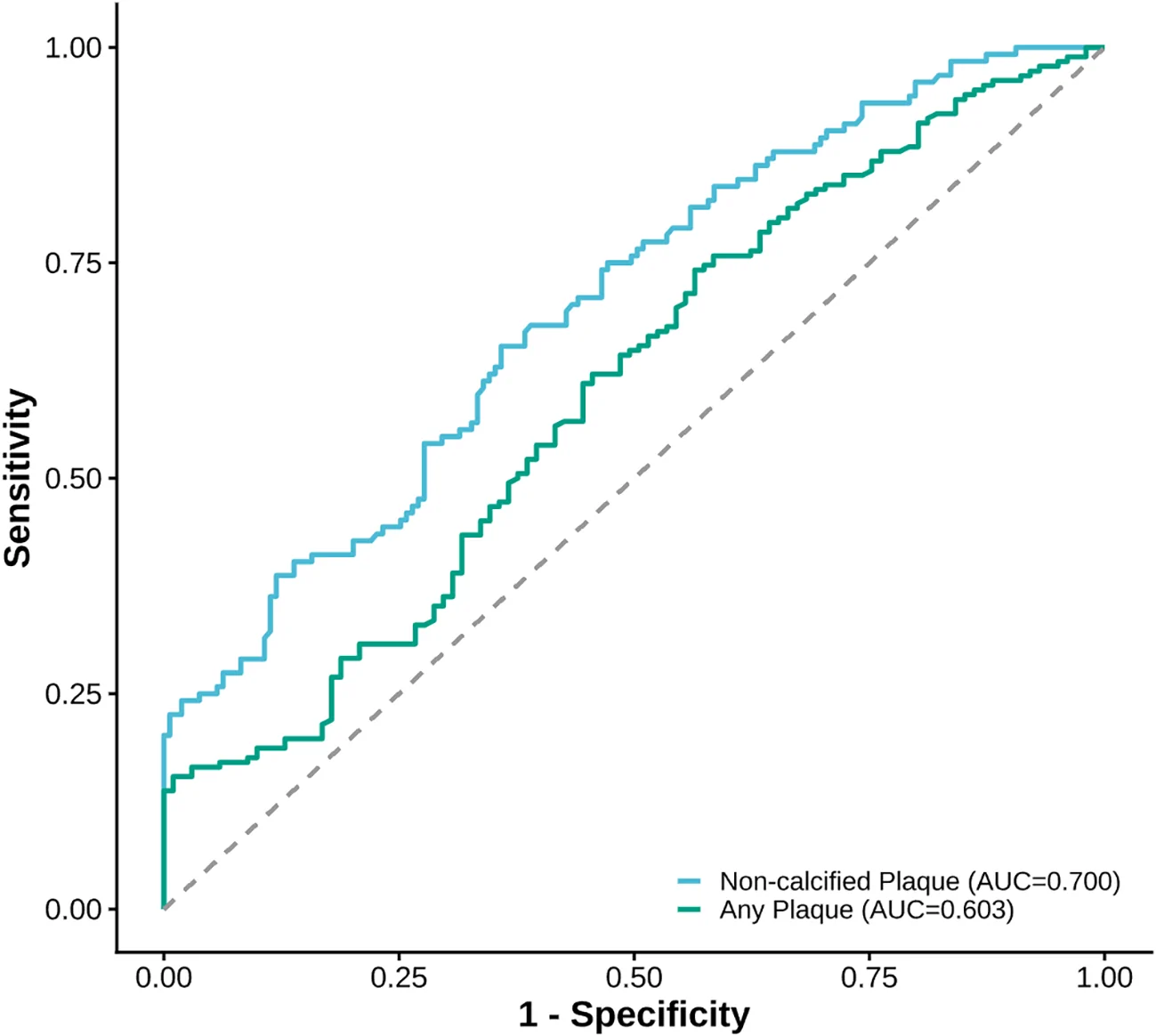
Receiver operating characteristic (ROC) curves of MHR for predicting coronary artery plaque.

**Table 4:** ROC curve analysis of MHR for coronary plaque detection.

Indicator	AUC	CI_95	P	Cutoff	Sensitivity	Specificity	Youden_Index
Non-calcified Plaque	0.700	0.640-0.761	<0.001	0.423	0.653	0.642	0.295
Any Plaque	0.603	0.534-0.672	0.004	0.343	0.742	0.436	0.177

P-value of < 0.05 was considered significant. MHR, Monocyte-to-HDL ratio; AUC, Area under the curve; CI, Confidence interval; ROC, Receiver operating characteristic.

To further explore the relationship between MHR and non-calcified plaque, we utilized restricted cubic spline (RCS) modeling. As shown in **Figure 2**, a significant non-linear association was observed (*P* for non-linearity = 0.007). The risk fluctuated across the MHR spectrum, showing a non-monotonic pattern rather than a simple linear increase.

**Figure 2:**
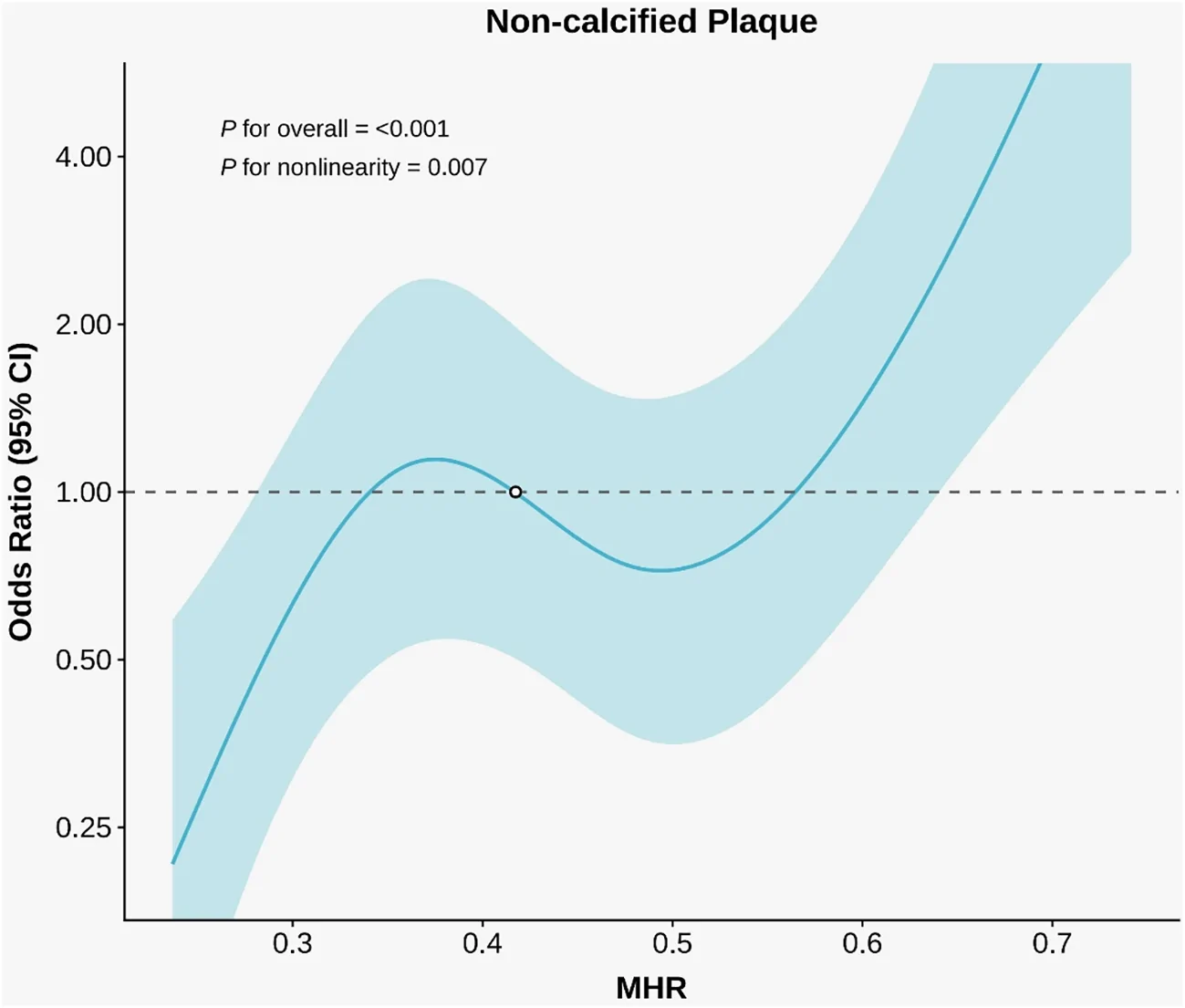
Non-linear association between MHR and non-calcified plaque risk by RCS.

### 3.5 Stratified analyses according to clinical characteristics

To assess the robustness of our findings and address potential interactions, subgroup analyses were performed based on a 1-SD increase in MHR (**Table 5**). The positive association between MHR and non-calcified plaque remained consistent across most stratified subgroups, including age (< 60 vs. ≥ 60 years), sex, diabetes status, smoking status, and BMI categories (< 25kg/m² vs. ≥ 25 kg/m²). For every 1-SD increase in MHR, the risk of non-calcified plaque increased approximately 2-fold in most subgroups. Although the association did not reach conventional statistical significance in the non-statin subgroup (OR = 1.77, 95% CI: 0.99–3.35, *P* = 0.063), the direction of the effect was consistent with that in the statin group, and no significant interaction was observed (*P* for interaction = 0.384). No significant interactions were observed between MHR and any of the stratifying variables (all *P* for interaction > 0.05), reinforcing the stability of MHR as an independent predictor in diverse clinical profiles.

**Table 5:** Subgroup analysis of the association between MHR and non-calcified plaque.

Variable	Subgroup	N	Events	OR	CI	P	P_Interaction
gender	Male	153	84	1.79	1.23-2.67	0.003	0.121
gender	Female	130	40	3.58	1.95-7.14	0.000	
age_group	<60 years	169	77	2.06	1.37-3.21	0.001	0.389
age_group	≥60 years	114	47	2.99	1.61-6.10	0.001	
diabetes	No	196	75	2.23	1.51-3.42	0.000	0.532
diabetes	Yes	87	49	2.76	1.48-5.71	0.003	
smoking_status	No	209	82	2.22	1.50-3.36	0.000	0.785
smoking_status	Yes	74	42	2.18	1.23-4.27	0.013	
bmi_group	< 25kg/m²	128	55	2.22	1.37-3.75	0.002	0.987
bmi_group	≥ 25 kg/m²	155	69	2.26	1.45-3.71	0.001	
statin	No	84	31	1.77	0.99-3.35	0.063	0.384
statin	Yes	199	93	2.29	1.56-3.46	0.000	

P-value of < 0.05 was considered significant. OR, Odds ratio; CI, Confidence interval. Model adjusted for age, sex, smoking status, BMI, triglycerides, LDL-C, serum creatinine, statin use, and diabetes (excluding the stratification variable).

## 4 Discussion

To our knowledge, this is the first study to examine the association between MHR and CCTA-based subclinical coronary atherosclerosis phenotypes specifically in asymptomatic patients with essential hypertension. The principal finding is that elevated MHR is independently associated with non-calcified coronary plaque prevalence, with evidence of a dose-response relationship. After adjusting for traditional cardiovascular risk factors including age, gender, diabetes, smoking, and LDL-C, patients in the highest MHR quartile (Q4) had a nearly 6-fold increased risk of harboring non-calcified plaques compared to those in the lowest quartile (Q1). ROC curve analysis demonstrated that MHR had moderate discriminatory ability for non-calcified plaque (AUC 0.700, 95% CI 0.640–0.761), with an optimal cutoff of 0.423 yielding a sensitivity of 65.3% and specificity of 64.2%. Restricted cubic spline analysis revealed a significant non-linear association, with an initial rise, a subsequent transient decline, and a steep increase at higher MHR levels. The RCS analysis complemented the quartile-based approach by uncovering a non-monotonic risk pattern that categorical analysis alone cannot capture. Subgroup analyses revealed no significant interaction between MHR and age, sex, diabetes, smoking status, BMI category, or statin use. Of note, given the limited statistical power of this single-center cohort, these negative findings should not be interpreted as evidence of absent effect modification. Interestingly, while MHR was strongly predictive of non-calcified plaques, its association with moderate-to-severe calcification, calcified plaques, mixed plaques, or significant luminal stenosis was attenuated and did not reach statistical significance in the fully adjusted model. This suggests that in the hypertensive population, MHR may specifically reflect the active, inflammatory phase of early atherogenesis rather than the later stages of stable calcification or fibrotic stenosis.

Prior studies have established MHR as a novel prognostic marker in patients with established coronary artery disease (CAD) and acute coronary syndromes, linking higher MHR to multivessel disease (9) and increased mortality (13). However, most of these studies focused on symptomatic patients with advanced disease. Our results focus on the subclinical stage, suggesting that MHR elevation is detectable at the subclinical stage. Our findings align with and extend prior evidence on MHR and coronary plaque characteristics. In a recent study of patients with psoriasis, Berna-Rico et al. demonstrated that MHR was independently associated with noncalcified coronary burden (NCB) and improved its prediction beyond traditional risk factors (AUC increased from 0.72 to 0.76), while no association with dense-calcified burden was observed (14). The present study extends this association to asymptomatic patients with essential hypertension, a population characterized primarily by hemodynamic stress–induced endothelial dysfunction rather than systemic autoimmune inflammation. Okan and Topaloglu demonstrated that MHR was significantly elevated in patients with unstable (mixed/soft) plaques compared with healthy controls, whereas no significant difference was observed between unstable and stable calcified plaque groups (15). These results conceptually align with our observation that MHR preferentially associates with non-calcified plaques. Conversely, Çelik et al. reported a positive association between MHR and coronary artery calcium score (CACS) in symptomatic patients (16), linking MHR to global calcification burden. While their findings appear to diverge from ours, this discrepancy likely reflects differences in study populations (symptomatic vs. asymptomatic). Our findings are further supported by studies on other metabolic indices. For instance, Park et al. reported that the Triglyceride-Glucose (TyG) index was significantly associated with non-calcified and mixed plaques but less so with calcified plaques in asymptomatic individuals (17). Similarly, Lee et al. found that elevated Lipoprotein(a) was an independent predictor of non-calcified plaque burden (18). Collectively, these findings suggest that MHR serves primarily as a marker of active, inflammatory plaque composition—specifically lipid-rich, soft plaques—rather than stable calcification. This biological specificity may arise from the dual nature of MHR, which simultaneously captures systemic inflammatory drive (monocytes) and impaired anti-atherogenic defense (HDL-C).

The distinct association between MHR and non-calcified plaques can be explained by the underlying pathophysiology of atherosclerosis, which is fundamentally a chronic inflammatory disease driven by lipid accumulation (19). Hypertension induces endothelial dysfunction and upregulates adhesion molecules, promoting the recruitment of circulating monocytes into the arterial intima (20). Once resident, monocytes differentiate into macrophages and avidly engulf oxidized LDL (ox-LDL) to form foam cells, which constitute the lipid-rich necrotic core characteristic of non-calcified plaques (19). Conversely, HDL-C exerts protective effects via reverse cholesterol transport and by inhibiting monocyte activation and LDL oxidation (21). A high MHR, therefore, indicates a “double hit” of enhanced pro-inflammatory drive and compromised anti-inflammatory defense. This imbalance fosters an active microenvironment that favors the formation and growth of soft, lipid-laden plaques. In contrast, calcified plaques are often considered to represent a more stable, healed stage of atherosclerosis or a response to chronic stress where inflammation has subsided (22). This mechanistic distinction likely explains why MHR strongly predicts non-calcified lesions while showing a weaker link to calcified burden in our multivariate analysis.

The clinical implications of identifying non-calcified plaques in asymptomatic hypertensive patients are substantial. Non-calcified plaques, particularly those with large lipid cores and thin fibrous caps, are prone to rupture and are the primary substrate for acute coronary syndromes (23). Conventional risk stratification often relies on identifying significant stenosis or heavy calcification (CACS), potentially missing these high-risk, non-obstructive soft plaques (24). Our study suggests that MHR, a widely available and cost-effective biomarker derived from routine complete blood counts, may serve as a valuable “gatekeeper” for risk stratification. While our study demonstrated a significant association between MHR and non-calcified plaques, the diagnostic performance yielded an AUC of 0.700, with an optimal cutoff of 0.423. It is important to distinguish the statistical meaning of this ROC-derived cutoff from the features observed in the restricted cubic spline (RCS) analysis. The ROC-identified cutoff represents the threshold that best separates individuals with and without non-calcified plaque in a binary classification context, maximizing sensitivity and specificity. In contrast, the RCS curve describes the continuous dose–response relationship and does not assign a single clinical decision point. The previously described non-monotonic pattern—an initial rise, a transient decline, and a steep subsequent increase—indicates that risk fluctuates across the MHR spectrum rather than increasing linearly above the cutoff. This distinction may explain why the optimal binary threshold (0.423) differs from the local curve features observed in the RCS analysis. Taken together, these findings suggest that MHR alone is insufficient as a standalone diagnostic tool for identifying specific plaque phenotypes. Importantly, the clinical utility of MHR does not lie in replacing advanced imaging modalities such as CCTA, but rather in functioning as a pragmatic screening biomarker. In primary care settings where asymptomatic hypertensive patients are routinely managed, an elevated MHR may serve as an adjunctive flag that, if prospectively validated, could help identify patients who may benefit from earlier risk factor modification or further imaging. Furthermore, it aids in triaging high-risk individuals who would derive the most benefit from subsequent CCTA screening.

## 5 Limitations

Several limitations of this study should be acknowledged. First, the cross-sectional design precludes causal inference; we cannot determine whether elevated MHR precedes plaque formation or is merely a marker of the disease process itself. Second, while we adjusted for multiple potential confounders, we lacked data on specific inflammatory cytokines (such as hs-CRP or IL-6) for direct comparison, and detailed information on the duration of antihypertensive or lipid-lowering therapies was not fully available for all patients. Third, our characterization of coronary plaques relied solely on conventional density thresholds to classify them as calcified, non-calcified, or mixed. We did not further evaluate specific high-risk plaque (HRP) features associated with vulnerability, such as low-attenuation plaque (<30 HU), positive remodeling, spotty calcification, or the napkin-ring sign. While non-calcified plaques are known to harbor lipid-rich or fibrous cores, not all non-calcified plaques are vulnerable. Future studies incorporating detailed HRP feature analysis are warranted to establish the relationship between MHR and true plaque vulnerability. Fourth, the study was conducted in a single center with a relatively small sample size, which may limit the generalizability of the findings to other ethnic or demographic groups, although the robustness of our results was supported by consistent subgroup analyses. Future large-scale, prospective longitudinal studies are needed to validate whether MHR-predicted non-calcified plaques translate into higher rates of major adverse cardiovascular events in this specific population. Fifth, these conditions were adjusted for using continuous measurements (BMI, lipid levels, and serum creatinine), which may not fully capture the presence of these comorbidities in treated patients whose values have been normalized by medication.

## 6 Conclusions

In summary, MHR is independently associated with the presence of non-calcified coronary plaques in patients with essential hypertension, with a significant non-linear dose-response relationship and consistent associations across diverse subgroups. Given the cross-sectional design, these findings cannot establish causality or predictive utility. Future prospective studies are needed to determine whether MHR can contribute to the identification of high-risk coronary phenotypes in this population.

## Data Availability

The raw data supporting the conclusions of this article will be made available by the authors, without undue reservation.
